# Factors Influencing COVID-19 Vaccine Confidence and Uptake in Australian Adults

**DOI:** 10.3390/vaccines12060627

**Published:** 2024-06-05

**Authors:** Charles Travers Williams, Bandana Saini, Syed Tabish R. Zaidi, Christina Kali, Grace Moujalli, Ronald Castelino

**Affiliations:** 1 Faculty of Medicine and Health, University of Sydney, Camperdown, NSW 2050, Australia; 2 School of Pharmacy and Pharmacology, College of Medicine and Health, University of Tasmania, Hobart, TAS 7005, Australia; 3 Pharmacy Department, Blacktown Hospital, Blacktown, NSW 2148, Australia

**Keywords:** vaccine hesitancy, COVID-19 vaccines, pandemic, immunisation, Australia, health knowledge, attitudes, practice

## Abstract

In January 2021, Australia initiated a national COVID-19 vaccine rollout strategy but faced setbacks, leading to negative press and media controversy, which may have diminished vaccine confidence. This study aimed to assess the factors influencing vaccine confidence in Australian adults (≥18 years of age) following the administration of a COVID-19 vaccine. Conducted at Blacktown Hospital, Sydney, a cross-sectional survey with 1053 respondents gauged vaccine confidence and influencing factors. The results showed overall high confidence (mean score 33/40). Trusted sources included the Australian Department of Health (77.8%), NSW Health (76.7%), and general practitioners (53.7%), while social media was distrusted (5.9%). The motivations for vaccination varied: university-educated individuals prioritised personal health (X^2^ = 17.81; *p* < 0.001), while religious and/or older respondents (≥50 years of age) emphasised community (X^2^ = 11.69; *p* < 0.001) and family protection (X^2^ = 17.314; *p* < 0.001). Multivariate logistic regression revealed use of the Australian Department of Health website as a trusted source of COVID-19 information as the strongest predictor of high confidence (>30; OR 1.43; *p* = 0.041), while exposure to fake news decreased confidence (OR 0.71; *p* = 0.025). The study underscores the importance of reliable health information sources in bolstering vaccine confidence and highlights the detrimental effects of misinformation. Promoting awareness of trustworthy health channels is crucial to combat vaccine hesitancy in Australia.

## 1. Introduction

The COVID-19 pandemic was undoubtedly one of the biggest global health crises in human history, disrupting societies and healthcare systems worldwide. In Australia, the onset of the pandemic was marked by the first confirmed case in Victoria, reported on the 25th of January, 2020 [1]. Since then, over 11 million COVID-19 cases have been reported nationwide, with more than 23,000 deaths as a direct consequence [2]—highlighting the severe public health impact of this disease, which is still ongoing as societies recover from economic and personal losses.

Although COVID-19 has now largely become an endemic disease, there remain many lessons from the pandemic period to be elucidated. At the time, in response to this global crisis, novel COVID-19 vaccines were being developed and approved at an unprecedented pace, and the Australian government, recognising the urgency of the situation, swiftly implemented the COVID-19 Vaccine National Rollout Strategy in January 2021, staggering the provision of vaccines by population group [3,4]. However, the rollout, while commendable in its intent, encountered significant challenges that ignited negative press and social media controversies [4,5].

In April 2021, the government-prioritised ChAdOx1-S vaccine was linked to a rare but serious safety signal of thrombosis with thrombocytopenia (TTS) [6], which led to intense negative media attention on the safety of the vaccine and the spread of vaccine misinformation on social media. With a higher risk of ChAdOx1-S-associated TTS suspected in younger adults, the BNT162b2 vaccine was recommended for all adults under the age of 50 [4,6]. This advice was revised in June 2021 and the age threshold raised to 60, following evidence of adults 50–59 years of age also being at higher risk of TTS with ChAdOx1-S. However, due to an insufficient supply of BNT162b2, healthy younger adults <40 years of age were ineligible to receive the vaccine until August 2021 in most parts of Australia [4,5]. Collectively, these challenges may have inadvertently contributed to diminished COVID-19 vaccine confidence and increased hesitancy among the Australian population.

Vaccine confidence is the belief in the effectiveness and safety of vaccines and trust in the system that delivers them [7]. It exists on a continuum, from no confidence to complete confidence, with multiple factors influencing an individual’s choice to receive a vaccine. Although distinct from vaccine hesitancy (i.e., the motivational state of being conflicted about or opposed to getting vaccinated), low vaccine confidence can contribute to hesitancy and both play a role in the decision to receive a vaccine and overall vaccine uptake [7].

The concept of vaccine confidence was first introduced in the early 21st century [8,9]. It emerged as a response to growing vaccine hesitancy, fuelled by misinformation and specific events that undermined public trust in vaccines, such as the now-debunked link between the measles, mumps, and rubella (MMR) vaccine and autism [10]. The establishment of the Vaccine Confidence Project (VCP) in 2010 and the focus of WHO’s Strategic Advisory Group of Experts (SAGE) on immunisation have been critical in defining and advancing the study of vaccine confidence [11,12]. The VCP was the first group to attempt to map vaccine confidence globally and found that the key reported reasons for vaccine hesitancy fell under the domains of “convenience”, “complacency”, and “confidence” [9]. In the recent 2022 VCP report commissioned by the EU, the results revealed that overall vaccine confidence had notably declined during the COVID-19 pandemic [13], which highlights the need to study the drivers behind these types of trends to help inform future pandemic vaccination strategies.

Understanding the factors affecting vaccine confidence is of paramount importance to support vaccine uptake from a public health perspective. Vaccines serve as the cornerstone for effective disease prevention and control, particularly in the context of contagious diseases, such as COVID-19. High levels of vaccine confidence within a population are pivotal for achieving high vaccine coverage and herd immunity, thereby reducing the transmission of disease and protecting those who cannot be vaccinated [14]. Conversely, low vaccine confidence can negatively impact uptake and may result in outbreaks, increased morbidity and mortality, and prolonged public health crises [15]. In an era where misinformation and scepticism can spread rapidly, the ability for government and public health bodies to instil vaccine confidence and support uptake is indispensable for safeguarding the health of communities and ensuring the collective well-being of the general population.

In Australia, some studies have evaluated the factors associated with COVID-19 vaccine hesitancy and the intention to vaccinate [16,17,18]. However, whether these factors lead to action and actual vaccination remains unknown. Limited evidence exists on the factors affecting vaccine confidence, especially the impact of media and “fake news”, and the motivators that result in vaccination in Australia. As such, the aim of this study was to determine the factors influencing COVID-19 vaccine confidence and uptake in Australian adults (≥18 years of age) following vaccine rollout changes.

## 2. Materials and Methods

### 2.1. Study Design and Data Collection

This cross-sectional study was conducted at a single centre between 4 August and 14 September 2021 during the expansion of COVID-19 BNT162b2 vaccine eligibility to the entire adult population in Australia [19].

All adults ≥18 years of age receiving their COVID-19 vaccinations at Blacktown Hospital, Sydney, were eligible to participate in this optional anonymised survey. Blacktown and Mount Druitt Hospital is a 534-bed tertiary teaching hospital with a large catchment area, caring for approximately 90,000 patients each year from culturally and linguistically diverse backgrounds in the Western suburbs of Sydney [20]. After receiving their vaccination (any dose), adults during the observation period were invited to complete a digital survey on their perspectives on COVID-19 vaccination by scanning a QR code located on posters in the waiting area using their mobile devices. The 10 min survey was administered online via the Research Electronic Data Capture (REDCap) tool, a secure web-based database application. A participation information sheet was available to all participants, and completion of the survey was considered implied consent.

The Human Research Ethics Committee and Western Sydney Local Health District granted approval to conduct this study (2021/ETH01038/STE02184).

### 2.2. Measures

A modified 22-item, two-part survey was utilised to assess vaccine confidence and influencing factors (see Supplementary File S1). Part 1 of the survey (14 items) assessed factors potentially associated with vaccine confidence and uptake and was adapted from the SAGE Working Group on Vaccine Hesitancy [12] and Centres for Disease Control (CDC) COVID-19 Vaccine Confidence: Rapid Community Assessment Tool [21]. The items assessed four domains: conditional (e.g., demographic, socioeconomic), social (e.g., sources of news and COVID-19 information), motivation (e.g., vaccination drivers), and practical influences (e.g., ease of access).

**Figure 1:**
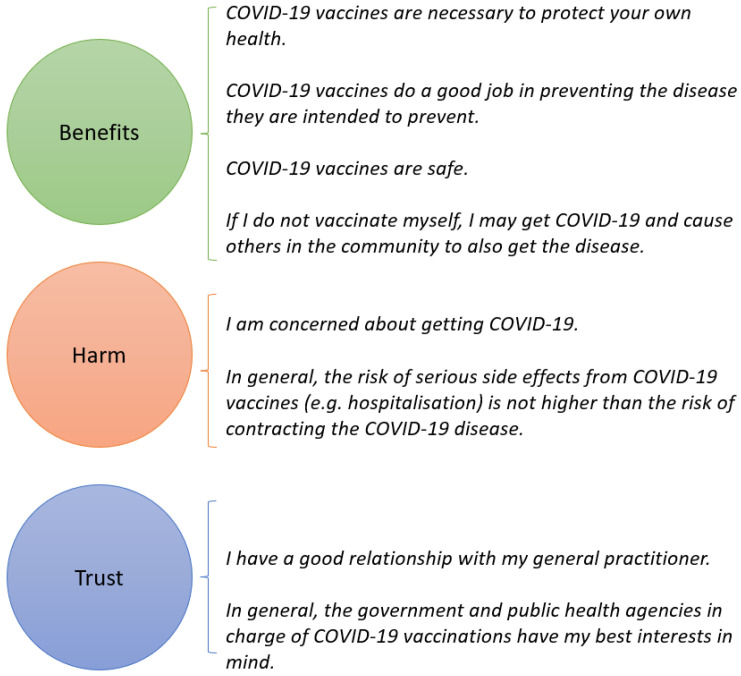
Part 2 of the survey: eight-item vaccine confidence scale.

In part 2 of the survey, COVID-19 vaccine confidence was measured using questions adapted from a validated vaccine confidence scale in parents of adolescents [22]. The 8 items of this scale were conceptualised using the Health Belief Model and adapted to fit the context of COVID-19 vaccinations in adults. The items used a 5-point Likert scale ranging from 1 (“strongly disagree”) to 5 (“strongly agree”) and assessed three areas integral to vaccine confidence and behaviour change: (1) Perceived benefits, (2) Perceived harm, and (3) Trust (see Figure 1). For the COVID-19 vaccine confidence scale, the total vaccine confidence score was calculated for each respondent (possible range 8–40) and stratified into low (≤20), medium (21–30), and high confidence (>30).

### 2.3. Data Analysis

Only eligible responses from adults ≥18 years of age were included as part of the final analyses. Descriptive statistics were used to describe the demographic characteristics, vaccine confidence, and frequency rates of conditional, social, motivational, and practical influences. Differences in demographics and factors that may influence motivators for vaccination were analysed using chi-square for discrete and non-normally distributed data and a *t*-test for continuous and normally distributed data. Confirmatory factor analysis was conducted for the adapted 8-item vaccine confidence scale to assess the model fit with the previously validated confidence scale used in adolescents (1-factor and 3-factor scales; “General Confidence” vs. “Benefits”, “Harm”, “Trust”) [22]. The comparative fit index (CFI) and root mean square of approximation (RMSEA) measures were used to assess the goodness of fit. An acceptable or “good fit” was defined as a CFI > 0.90 and RMSEA < 0.08 [23,24]. A chi-square goodness-of-fit test was also conducted for the entire sample to assess the model’s distribution uniformity. To check the scale reliability, Cronbach’s alpha coefficients were calculated for the “General Confidence” total scale and the subscales (“Benefits”, “Harm”, and “Trust”), with acceptable reliability considered α ≥ 0.7 [25]. For complete data, the relationship between high COVID-19 vaccination confidence and potential predictors was analysed using univariate and multivariate logistic regression with odds ratios to measure the effect size. Variables with a statistically significant association in the univariate analysis were included in the multivariable logistic regression model. The statistical analyses were performed using IBM SPSS (version 28.0) with all significance levels set at *p* < 0.05.

## 3. Results

The survey was completed by 1060 respondents. Of those, 7 adolescent respondents were excluded, with 1053 included in the final analysis (Table 1), and 1011 had complete data (96.02%). The mean age of the respondents was 36.7 years (standard deviation [SD] 10.0), with 71.0% of them between 18 and 40 years of age. Slightly more respondents were male (51.5%), and the majority had an education level of undergraduate level or higher (78.5%). Most of the respondents identified themselves as of Indian (40.8%) and/or Australian (28.8%) ancestry, with Hinduism (33.1%), Christianity (28.9%), and atheism (21.5%) as the most cited religious beliefs. The distribution of the reported household income was negatively skewed, with most respondents on a weekly income of >$1500 AUD per week (55.8%). Among the respondents, 21.9% had a medical condition or risk factor associated with a high risk of severe COVID-19; the most common were asthma (26.4%), current smoking status (17.8%), and obesity (14.7%). A healthcare professional recommendation to receive a COVID-19 recommendation was provided to 30.1% of the respondents, and 20.1% had experienced COVID-19 disease, either personally or via a family or friend. 

### 3.1. Sources of News and COVID-19 Information

**Figure 2:**
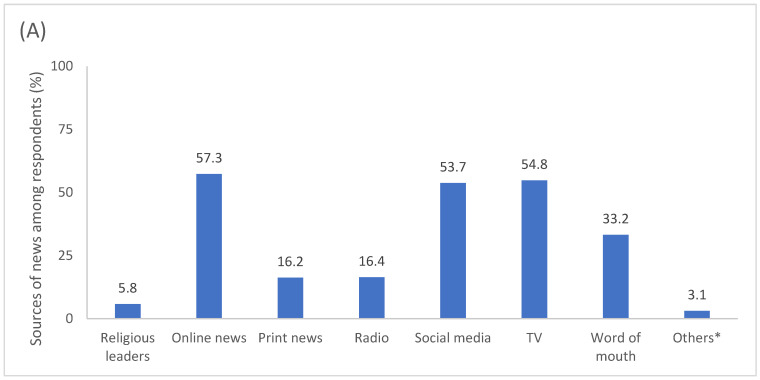

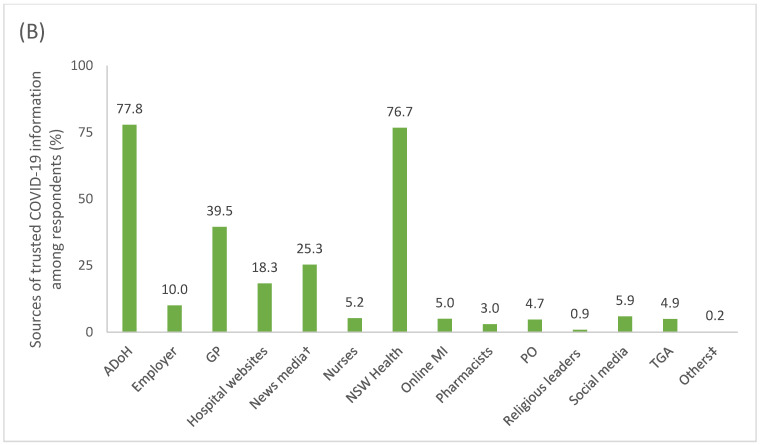
(**A**) Sources of news and (**B**) trusted sources of COVID-19 information reported among respondents. * Other sources of news included YouTube and podcasts. † Television, internet, radio. ‡ Other trusted sources of COVID-19 information included WHO, CDC, overseas healthcare bodies, friends, and family. ADoH, Australian Department of Health; CDC, Centers for Disease Control and Prevention; GP, general practitioner; MI, medical information; NSW, New South Wales; PO, professional organisations, TGA, therapeutic goods administration; WHO, World Health Organization.

The reported sources of news and trusted sources of COVID-19 information are provided in Figure 2. Among the respondents, the most frequent sources of news were online news articles (*n* = 603; 57.3%), TV (*n* = 577; 54.8%), and social media (*n* = 565; 53.7%). For COVID-19 information, the top three trusted sources were the Australian Department of Health (ADoH; *n* = 819; 77.8%), New South Wales Health (NSWH; *n* = 808; 76.7%), and general practitioners (*n* = 416; 39.5%). The news media were considered a trusted source of COVID-19 information by 25.3% (*n* = 266) of the respondents. In comparison, social media was a trusted source for only 5.9% (*n* = 62) of the respondents. The other reported trusted sources of COVID-19 information, including other healthcare professionals (e.g., nurses and pharmacists), were reported at a low level (≤10%). When the respondents were asked whether they had seen or heard any information about COVID-19 vaccines for which they could not determine whether it was true or false, more than half (*n* = 606; 57.5%) were found to have potentially been exposed to “fake news” or misinformation.

### 3.2. Motivators for Receiving a Vaccination

**Figure 3:**
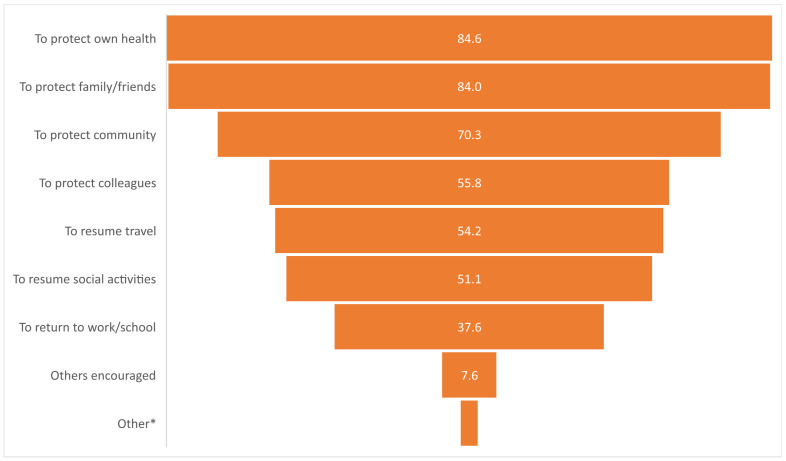
Reported motivators for receiving a COVID-19 vaccination among respondents (%). * Other common reported motivators included pregnancy/to protect their baby, healthcare professional recommendation, and to support the economy.

The reported motivators among the respondents for receiving a COVID-19 vaccination are outlined in Figure 3. The most common reported motivator for receiving a COVID-19 vaccination was to protect one’s health (84.6%; *n* = 891), followed by the protection of family and friends (84.1%; *n* = 885) and the community (70.3%; *n* = 740). Respondents with university degrees were more likely to be motivated by the protection of their health (X^2^ = 17.81; *p* < 0.001), while those who reported being religious were more likely to be motivated to protect their community (X^2^ = 11.69; *p* < 0.001). Similarly, respondents aged 50 years and older were more likely to be motivated to receive a COVID-19 vaccination (X^2^ = 17.314; *p* < 0.001) by the notion of protecting their family and friends.

### 3.3. Predictors of High Vaccine Confidence

The overall vaccine confidence scores were high, with a mean score of 33.0 (SD 5.24) and with 3.0% (*n* = 32), 21.5% (*n* = 226), and 75.5% (*n* = 795) of the respondents categorised as having low, medium, and high vaccine confidence, respectively. Most of the respondents agreed with the perceived benefits of COVID-19 vaccines and the perceived harm of contracting COVID-19 and trusted the healthcare system and government (see Supplementary Figures S1–S3). When comparing the mean scores across the perceived Benefits, Harm, and Trust domains, the respondents generally gave higher ratings to Benefits (four items, mean 4.25; standard error [SE] 0.02) than to Harm (two items, mean 3.98; SE 0.03) and Trust (two items, mean 4.03; SE 0.02).

For the one-factor scale (General Confidence), the model showed a partially acceptable fit (see Supplementary Figure S4), with a CFI = 0.95 and X^2^(20) = 166.6 (*p* < 0.001) but a RMSEA = 0.084. The standardised factor loadings ranged from 0.33 to 0.84 (Table S1). For the three-factor scale (Benefits, Harm, and Trust), the model showed a good fit, with a CFI = 0.97, RMSEA = 0.071, and an X^2^(17) = 105.9 (*p* < 0.001). The standardised factor loadings for the Benefits, Harm, and Trust subscales ranged from 0.67 to 0.86, 0.40 to 0.69, and 0.42 to 0.77, respectively. Good internal scale consistency was demonstrated, with an overall Cronbach’s alpha coefficient of 0.82 (Benefits α = 0.84; Harm α = 0.43; Trust α = 0.49).

The initial univariate analysis identified seven predictors significantly associated with high vaccine confidence (total score > 30; see Table 2). Having a university degree (OR 1.43; *p* = 0.034) and the use of the ADoH (OR 1.64; *p* = 0.003) or NSWH (OR 1.46; *p* = 0.021) as trusted sources of COVID-19 information were associated with higher vaccine confidence among the respondents. In contrast, older age (OR 0.99; *p* = 0.036), Australian ethnicity (OR 0.72; *p* = 0.037), social media as a trusted source of COVID-19 information (0.51; *p* = 0.015), and exposure to fake news (OR 0.71; *p* = 0.024) negatively impacted vaccine confidence. Following the multivariate analysis, the strongest predictor of high vaccine confidence was the use of the ADoH (OR 1.43; *p* = 0.041) as a trusted source of COVID-19 information, while exposure to fake news was the strongest negative predictor (OR 0.71; *p* = 0.025). No other predictors reached statistical significance.

## 4. Discussion

In the present study, the factors influencing COVID-19 vaccine confidence and uptake in Australian adults (≥18 years of age) were assessed using a cross-sectional survey conducted at Blacktown Hospital following COVID-19 vaccination. The study found that the overall vaccine confidence was high and that the use of government websites, such as the ADoH, as sources of COVID-19 information resulted in higher vaccine confidence among the respondents, whereas exposure to social media appeared to undermine confidence.

Personal and community safety perspectives were key motivations in the sample of respondents. Those receiving a vaccine were often motivated to get vaccinated to protect their own health, the health of their friends and family, and/or their community (70.3–84.6%). Similar results were reported in an international cross-sectional study conducted in Australia, Norway, the UK, and the US (2021–2022), which found that among those who had received a COVID-19 vaccine (*n* = 1396), the most common motivation reasons were to reduce their risk of illness (86%) and for the health of others (74%) [26]. Likewise, a survey of Australian adults in 2022 found that among those classified as vaccine-acceptant (*n* = 353), protecting oneself and others was the key reason for receiving the primary vaccination series [27]. Interestingly, in this study, respondents who were older (≥50 years of age) or religious were more likely to be motivated by altruistic notions of protecting others, while those with university degrees were more motivated by the protection of themselves. The results highlight how health information and vaccine recommendations could potentially be tailored in order to improve vaccine confidence and uptake.

In this study, as the respondents had already chosen to receive a COVID-19 vaccination, the high overall vaccine confidence reported was expected. A US study that used a similar eight-item vaccine confidence scale saw similar results in parents with established adolescent vaccines (mean total vaccine confidence score 33.0 in the present study vs. 32.6 in the US study) [28]. As COVID-19 vaccines were novel at the time of the survey, it appears that increasing vaccine confidence levels to those seen for more established well-known vaccines may be critical in supporting the acceptance and uptake of newer vaccines. This is supported by a large retrospective study of global trends in vaccine confidence using data from 290 surveys conducted in 149 countries from 2015 to 2019, which found that high vaccine confidence was the most consistent determinant associated with improved vaccine uptake [29]. Similarly, in another cross-sectional online survey (*N* = 1166) conducted in Australia that evaluated vaccine confidence as a secondary outcome in February 2011 before the ChAdOx1-S vaccine controversy, most of the respondents (>60%) agreed on the safety, importance, and effectiveness of general vaccines [16]. Strong agreement with general vaccine safety, importance, and effectiveness among the respondents was associated with a higher likelihood of COVID-19 vaccine uptake (OR 14.0; 95% CI 10.4–18.9).

Interestingly, the eight-item scale used to assess COVID-19 vaccine confidence in this study appeared to offer an efficient measure of adult vaccination beliefs. The scale, adapted from Gilkey et al.’s scale validated in parents of adolescents, had similar confirmatory factor analysis results and fit the data best when divided into a three-factor scale assessing the perceived benefits of vaccination, harm of contracting COVID-19, and trust in the healthcare system and government [22,28]. The adapted vaccine confidence scale also demonstrated good internal consistency, with an overall Cronbach’s alpha coefficient of 0.82 (Benefits α = 0.84; Harm α = 0.43; Trust α = 0.49). Cronbach’s alpha coefficient previously reported in the study by Gilkey et al. was 0.77 (Benefits α = 0.78; Harm α = 0.49; Trust α = 0.51) [28]. By evaluating the domains of Benefits, Harms, and Trust, the vaccine confidence scale may guide interventions and communications intended to enhance adult receptiveness to vaccination. However, future studies in different settings are warranted to further assess the validity of the scale in adults.

When exploring the domains that contribute to overall vaccine confidence, the respondents reported higher scores for the perceived Benefits of COVID-19 vaccines compared with those for the Harm and Trust domains (mean 4.25 vs. 3.98 and 4.03, respectively). The perceived Benefit results were largely consistent with those reported in parents with established adolescent vaccines (4.23; SE 0.03); however, the Harm score was higher (3.33; SE 0.04) and the Trust score lower (4.52; SE 0.02) than those previously reported in parents with adolescent vaccines [28]. This was likely due to the COVID-19 pandemic situation at the time in Australia, whereby the number of reported cases was increasing steadily [11] and the vaccine rollout had just recovered from some highly publicised setbacks [4]. Furthermore, in a 2021 cross-sectional survey study of older adults (≥70 years of age) and adults with underlying conditions (18–69 years of age) in Victoria, Australia, the benefits of vaccination, specifically the efficacy and safety of the vaccine, were significantly associated with the intention to accept a COVID-19 vaccine [18]. Collectively, this suggests that although all three domains are important for high vaccine confidence, the perceived benefit of a vaccine may be critical to actual vaccine uptake. When considering immunisation campaign messaging, appropriate emphasis should be placed on the benefits of vaccination and supported by trustworthy sources. This approach would likely improve the effectiveness of campaign communications and aid the public in making informed decisions about vaccinations.

Predictors of high vaccine confidence (total score > 30) initially identified through univariate analyses were having a university degree and the use of government sources of trusted health information (ADoH and NSW health). Meanwhile, older age, being of Australian ethnicity, the use of social media as a trusted source of COVID-19 information, and exposure to fake news were factors associated with a lower likelihood of high vaccine confidence. Following the multivariate analysis, only two factors remained, with the use of the ADoH as a trusted source of COVID-19 information increasing the likelihood of high vaccine confidence by 43% (OR 1.43; 95% CI 1.02–2.00) and exposure to fake news decreasing the likelihood by 29% (OR 0.71; 95% CI 0.52–0.96). These results were largely consistent with other COVID-19 vaccine confidence studies. A cross-sectional survey study conducted in New Zealand (Aug–Oct 2021; *N* = 1852) reported that COVID-19 vaccine confidence increased with increasing educational levels (*b* = 0.06; *p* < 0.01), as well as with age (*b* = 0.16; *p* < 0.001) [30]. Another cross-sectional survey study from Canada (Oct–Nov 2020; *N* = 1599) found that having a university degree was significantly associated with a high level of vaccine confidence (OR 2.14; 95% CI 1.40–3.30). Having parents from Canada (OR 2.18; 95% CI 1.35–3.52) and an older age (≥60 years of age, OR 3.30; 95% CI 2.13–5.13) were also significantly associated with high vaccine confidence [31]. Differences in the results may be explained by differences in the study methodology and vaccine confidence assessment. Additionally, the unique challenges faced in the rollout of COVID-19 vaccines for adults in Australia and the negative media attention at the time surrounding ChAdOx1-S-associated TTS adverse events, with limited vaccine alternatives for older adults, may have also contributed to lower vaccine confidence among older adults and Australians.

Although not identical to vaccine confidence measures, the results from previous vaccine hesitancy studies on COVID-19 vaccines in Australia were comparable. A longitudinal survey study (Feb–Aug 2020; *N* = 3061) found that individuals with undergraduate or postgraduate degrees were less likely to be vaccine-resistant or hesitant compared with those with Year 12 education only [32]. Moreover, those who had confidence and trust in their state or the government or confidence in their hospitals were more likely to get vaccinated. Similar findings were reported in a cross-sectional survey study by Wang et al. [17].

Regarding the impact of media and exposure to “fake news”, multiple studies have shown that the media can negatively impact vaccine confidence and increase vaccine hesitancy [33,34,35]. For example, Catalan-Matamoros et al. reported a significant inverse correlation between negative newspaper coverage and childhood vaccination rates in Spain (r = −0.771; *p* < 0.05) [33]. Similarly, Suppli et al. found a 36% drop in HPV vaccination uptake in girls born in Denmark in 2003 following a spike in negative media coverage [35]. This study further supports the negative impact of misinformation on vaccine confidence and its potential consequences for vaccine uptake. The dissemination of healthcare information through trusted sources of information, such as government or non-profit organisations, is crucial, especially during disease outbreaks. In a recent study of 23 government websites, only 65.4% had dedicated information on COVID-19 [36]. Likewise, only 64.71% of government websites provided exclusive communication channels for COVID-19-related queries, highlighting the opportunity to increase the frequency of government communications on healthcare and vaccines. Furthermore, the general population often struggles to discern between what is true and what is false healthcare information, and this is often linked to low health literacy. In Australia, 60% of people are reported to have low health literacy [37]. This study found that more than 1 in 2 of the respondents (57.5%) reported that they had been exposed to COVID-19 information that they were unable to determine the validity of. As such, further research is needed to develop improved healthcare communications on vaccines and support the public’s ability to identify trusted sources of information and discern when they may be receiving misinformation. A recent US study by Hurstak et al. (*N* = 271) found that both high vaccine confidence and high health literacy were associated with COVID-19 vaccination and that vaccine confidence mediated the relationship between the three (mediator effects: 0.04; 95% CI 0.02–0.08) [38]. There is also emerging research on theory-informed information debiasing interventions, such as debunking, that can be used to dispel misinformation [39]. Such interventions need to be developed perhaps specifically to address vaccine misinformation, and this research is critical for future pandemic management.

To the best of our knowledge, this is the only study that has measured COVID-19 vaccine confidence and assessed the motivators for vaccination immediately following immunisation in Australia. While this study adds to the evidence on the factors associated with high vaccine confidence and motivators for receiving a COVID-19 vaccination, several limitations exist. (1) The study population was specific to a single centre in Australia and COVID-19 primary series vaccination, which may limit the generalisability. (2) This study was cross-sectional and observational; thus, no causal links can be established. Longitudinal data are required to identify whether the predictors of high vaccine confidence are maintained and whether they apply to COVID-19 boosters. (3) Assessment of the type of COVID-19 vaccine received was not assessed, and vaccine confidence may vary between vaccines. However, at the time, access to alternative vaccines was limited, and most were likely to have received the BNT162b2 vaccine. Moreover, the series dose, history of previously received vaccines, and whether a respondent was a healthcare professional were not captured and may have resulted in residual confounding. (4) Finally, the survey was conducted in individuals who had already made the decision to receive a COVID-19 vaccine, and as such, the overall vaccine confidence was generally high. Although this benefits the identification of true motivators for vaccination, non-normal data distributions may introduce bias into the estimates of statistical measures. However, a histogram of the overall vaccine confidence scores revealed only minimal skewing, suggesting a limited impact to statistical analyses (Figure S5). Nevertheless, further research is warranted in the general population who have not yet received a COVID-19 vaccine to verify factors associated with high vaccine confidence and to further determine how media may impact overall vaccine confidence and the intent to get vaccinated.

## 5. Conclusions

Vaccine confidence was high in individuals receiving a COVID-19 vaccine in Australia despite changes to the national vaccine rollout strategy. Individuals who received vaccine information from government health sites were significantly more likely to have high vaccine confidence. On the contrary, exposure to “fake news” or health information that individuals could not determine to be true or false negatively impacted vaccine confidence. Given the potential negative effects of misinformation on vaccine confidence, improved education on tackling disinformation and higher awareness of trusted sources of health information among the general public are needed.

## Figures and Tables

**Table 1:** Respondent demographics.

*N* = 1053	*n*	%
**Gender**		
Female	509	48.3
**Age**		
18–24	105	10.0
25–34	340	32.3
35–44	408	38.7
45–54	122	12.0
55–64	55	5.2
65+	12	1.1
**Ancestry**		
Aboriginal/Torres Strait Islander	3	0.3
Australian	303	28.8
Chinese	81	7.7
English	37	3.5
Filipino	63	6.0
German	2	0.2
Indian	430	40.8
Irish	13	1.2
Italian	11	1.0
Other ancestry *	194	18.4
Scottish	9	0.9
**Religion**		
Buddhism	32	3.0
Christian	304	28.9
Hinduism	349	33.1
Islam	86	8.2
No religion	226	21.5
Other religion ^†^	24	2.3
Sikhism	25	2.4
**Education**		
Less than high school	8	0.8
High school	165	15.7
Bachelor’s degree	469	44.5
Master’s degree	340	32.3
PhD or higher	18	1.7
Trade school	48	4.6
**Income**		
$1–500 per week	90	8.5
$501–1000 per week	161	15.3
$1001–1500 per week	203	19.3
$1501–2000 per week	181	17.2
$2001–2500 per week	150	14.2
>$2500 per week	257	24.4
**Medical condition assoc. high risk of severe COVID-19** ^‡^		
Yes	231	21.9
**HCP recommended the vaccine**		
Yes	317	30.1
**Previous COVID-19 experience ^§^**		
Yes	212	20.1

* Most common other ancestries were Pakistani (2.6%), Iranian (1.4%), Nepalese (0.9%). ^†^ Most common other religions were Jainism (0.6%) and Judaism (0.2%). ^‡^ Asthma (26.4%), cancer (2.16%), smoker (17.8%), CKD (1.7%), COPD (0.4%), cystic fibrosis (0.9%), diabetes (10.0%), heart disease (3.5%), hypertension (13.9%), IC/IS (3.0%), liver disease (1.3%), obesity (14.7%), previous stroke (0.9%), solid organ or blood stem cell transplantation (0.4%), thalassemia (3.0%). ^§^ The respondent, someone in their family, or a friend had contracted COVID-19.

**Table 2:** Predictors of high vaccine confidence (total score > 30).

	Univariate Analysis			Multivariate Analysis		
	OR	95% CI	*p* Value	OR	95% CI	*p* Value
Age	0.99	0.97–0.99	0.036	0.99	0.97–1.00	0.107
Australian	0.72	0.53–0.98	0.037	0.78	0.56–1.09	0.146
University degree	1.43	1.03–2.00	0.034	1.18	0.82–1.71	0.372
ADoH	1.64	1.18–2.27	0.003	1.43	1.02–2.00	0.041
NSWH	1.46	1.06–2.02	0.021	1.33	0.95–1.87	0.093
Social media	0.51	0.30–0.88	0.015	0.60	0.34–1.05	0.075
Exposure to fake news	0.71	0.53–0.96	0.024	0.71	0.52–0.96	0.025

ADoH, Australian Department of Health; CI, confidence interval; NSW, New South Wales Health; OR, odds ratio.

## Data Availability

The data presented in this study is available on request from the corresponding author.

## Supplementary Materials

The following supporting information can be downloaded at https://www.mdpi.com/article/10.3390/vaccines12060627/s1. File S1: Factors that may influence vaccine confidence survey; Appendix Figure S1: Perceived benefits: distribution of Likert score results from respondents agreeing or disagreeing with the four statements pertaining to the benefits of COVID-19 vaccination; Figure S2: Perceived harm: distribution of Likert score results from respondents agreeing or disagreeing with the four statements pertaining to the harm of COVID-19 disease; Figure S3: Trust: distribution of Likert score results from respondents agreeing or disagreeing with the four statements pertaining to the trust of healthcare systems and government agencies; Figure S4: Confirmatory factor analysis of the vaccine confidence one-factor and three-factor scales with standardised factor loading values; Figure S5: Histogram of overall vaccine confidence scores; Table S1: Vaccine confidence one- and three-factor scale item mean scores and factor loadings.
