# Supplementary material for: Factors Influencing COVID-19 Vaccine Confidence and Uptake in Australian Adults
Source: Vaccines (Basel). 2024 Jun 5;12(6):627. doi: 10.3390/vaccines12060627 (PMC11209045; doi:10.3390/vaccines12060627)
Supplement: Supplementary file 1 [file vaccines-12-00627-s001.zip › Supplementary appendix.pdf]

Supplementary appendix.

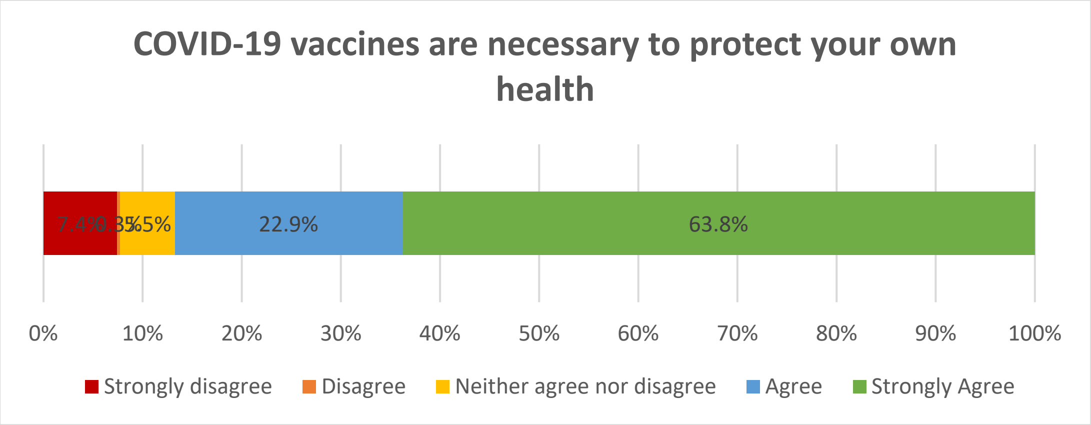

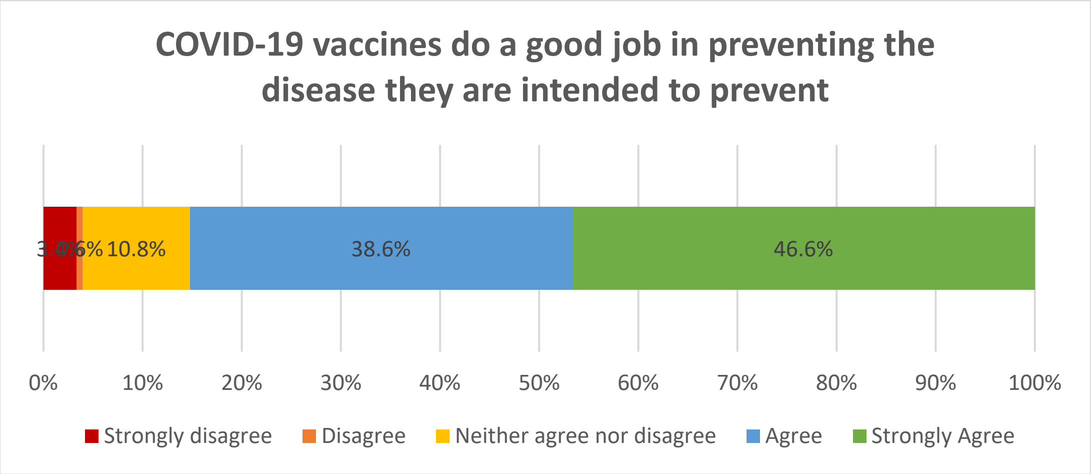

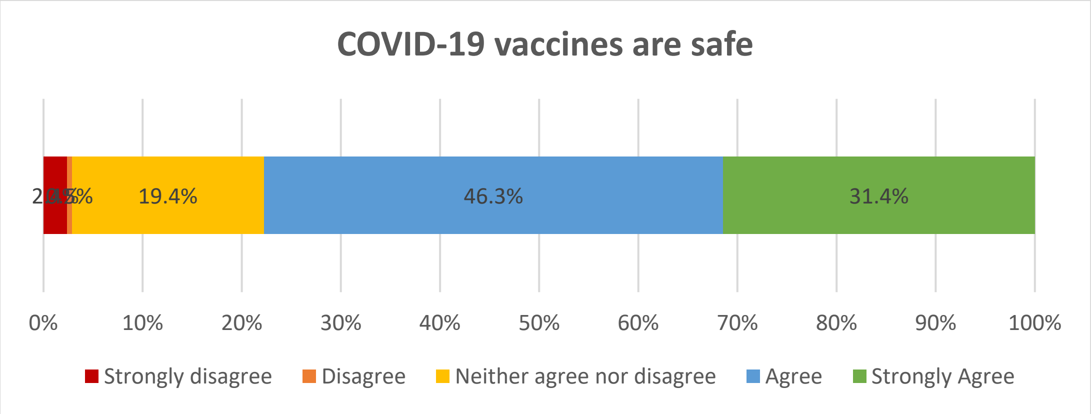

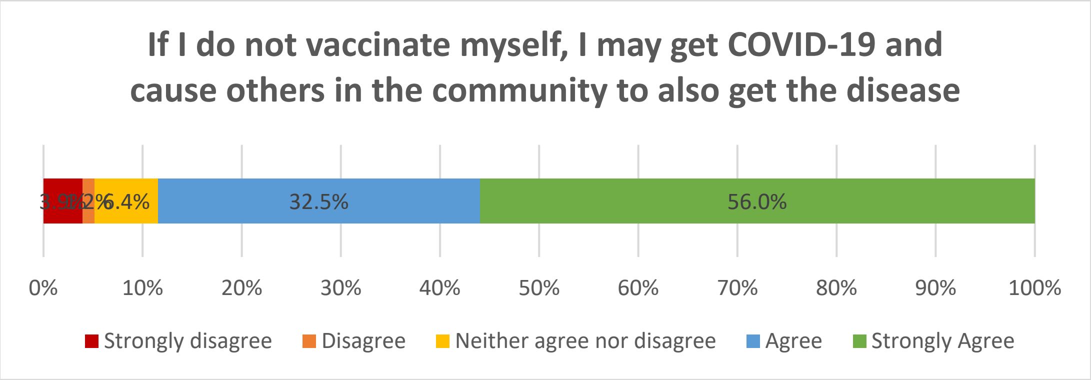

Figure S1. Perceived benefits: distribution of Likert score results from respondents agreeing or disagreeing with the four statements pertaining to the benefits of COVID-19 vaccination.

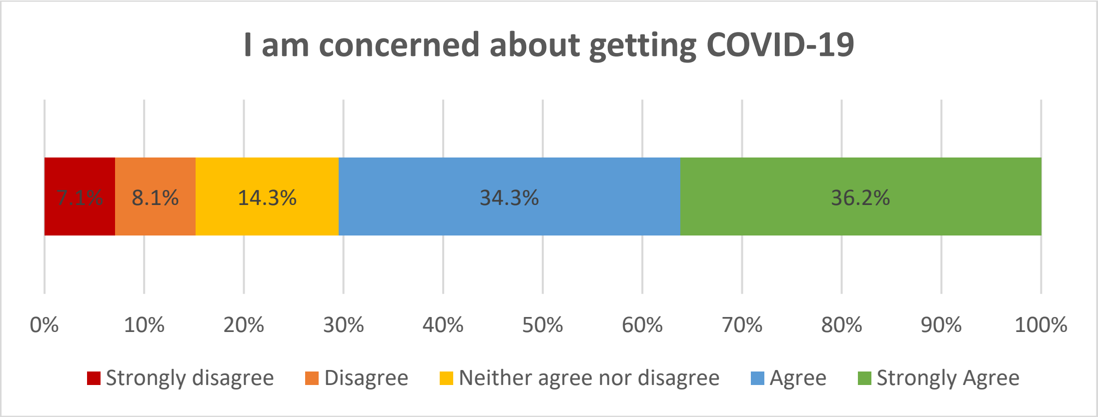

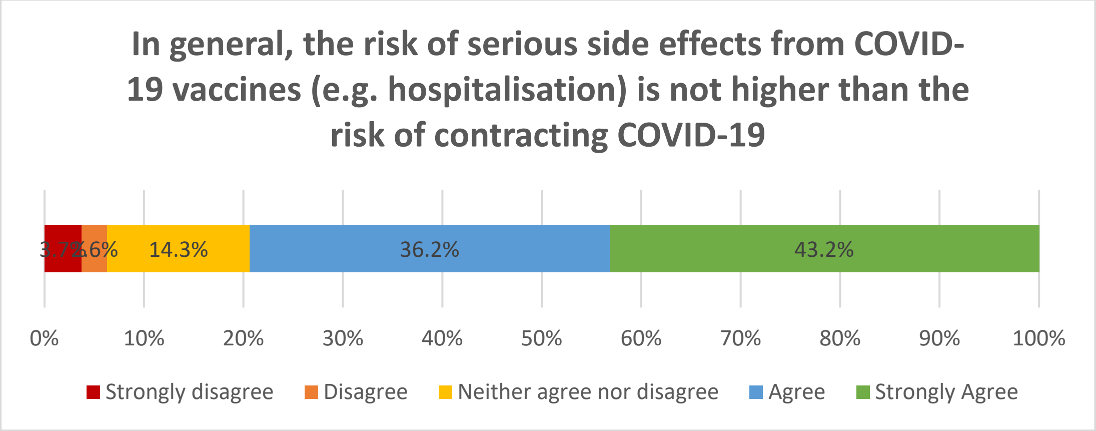

Figure S2. Perceived harm: distribution of Likert score results from respondents agreeing or disagreeing with the four statements pertaining to the harm of COVID-19 disease.

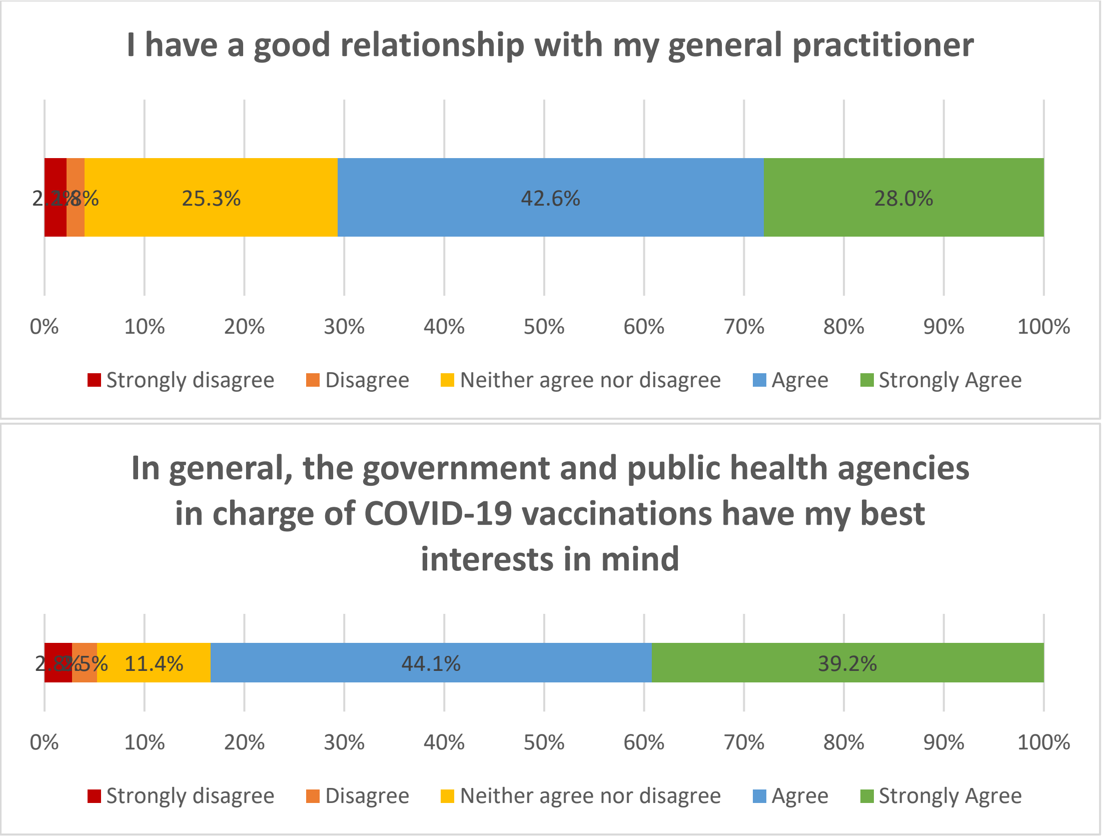

Figure S3. Trust: distribution of Likert score results from respondents agreeing or disagreeing with the four statements pertaining to the trust of healthcare systems and government agencies.

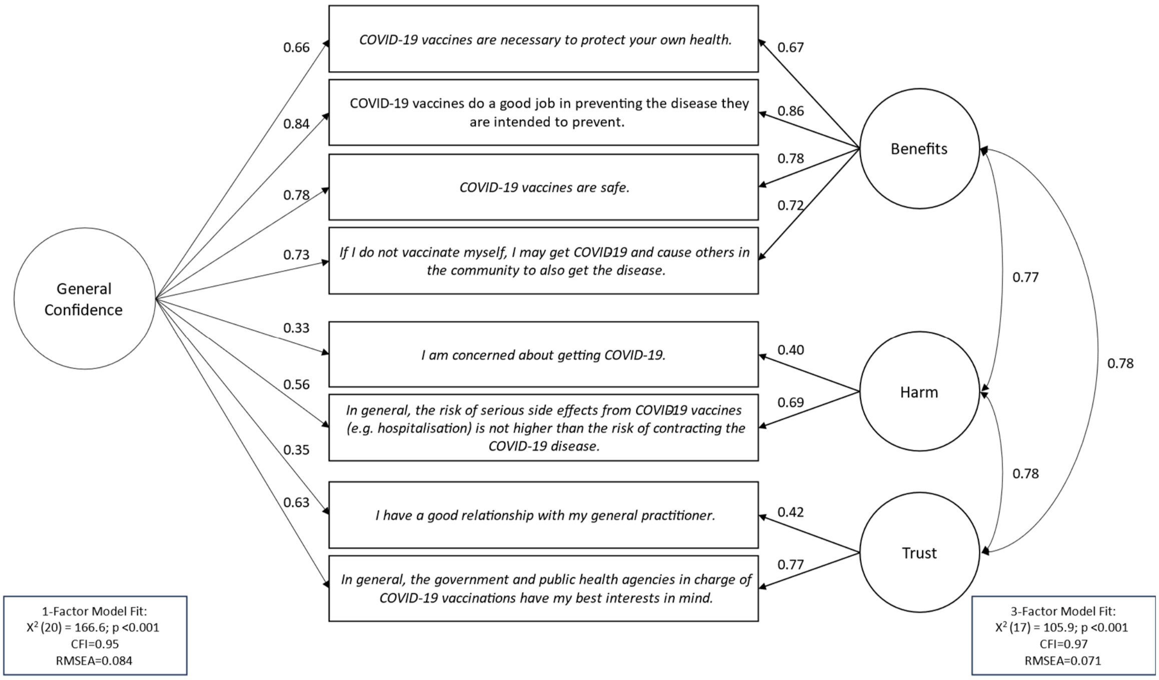

Figure S4. Confirmatory factor analysis of the Vaccine Confidence 1-factor and 3-factor scales with standardised factor loading values

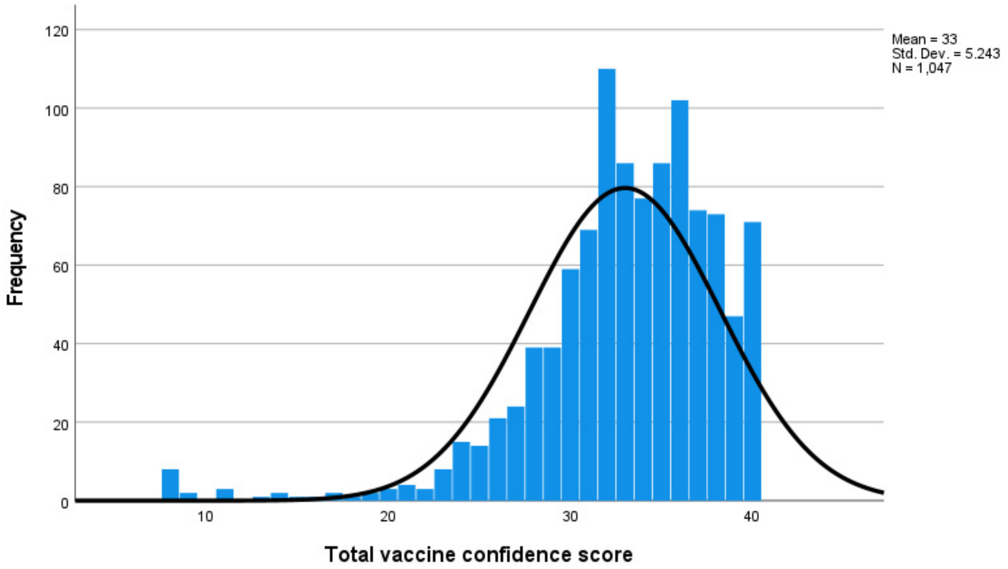

Figure S5. Histogram of overall vaccine confidence scores.

Table S1. Vaccine confidence 1- and 3-factor scale item mean scores and factor loadings

8 items	Item Mean (SE)	Standardised factor loading (SE)			
		1-Factor	3-Factor Scale		
		General confidence	Benefits	Harm	Trust
COVID-19 vaccines are necessary to protect your own health.	4.36 (0.04)	0.66 (0.03)	0.67 (0.03)	-	-
COVID-19 vaccines do a good job in preventing the disease they are intended to prevent.	4.25 (0.03)	0.84 (0.02)	0.86 (0.02)	-	-
COVID-19 vaccines are safe.	4.04 (0.03)	0.78 (0.02)	0.78 (0.02)	-	-
If I do not vaccinate myself, I may get COVID-19 and cause others in the community to also get the disease.	4.36 (0.03)	0.73 (0.02)	0.72 (0.02)	-	-
I am concerned about getting COVID-19.	3.84 (0.04)	0.33 (0.06)	-	0.40 (0.06)	-
In general, the risk of serious side effects from COVID-19 vaccines (e.g. hospitalisation) is not higher than the risk of contracting the COVID-19 disease.	4.12 (0.03)	0.56 (0.03)	-	0.69 (0.05)	-
I have a good relationship with my general practitioner.	3.92 (0.03)	0.35 (0.03)	-	-	0.42 (0.03)
In general, the government and public health agencies in charge of COVID-19 vaccinations have my best interests in mind.	4.14 (0.03)	0.63 (0.02)	-	-	0.77 (0.04)
Cronbach alpha coefficient		0.82	0.84	0.43	0.49

SE, standard error
