# Supplementary material for: Factors Influencing COVID-19 Vaccine Confidence and Uptake in Australian Adults
Source: Vaccines (Basel). 2024 Jun 5;12(6):627. doi: 10.3390/vaccines12060627 (PMC11209045; doi:10.3390/vaccines12060627)
Supplement: Supplementary file 1 [file vaccines-12-00627-s001.zip › Supplementary file.pdf]

Factors that may influence vaccine confidence

1. **What gender do you identify as? [C]**
    a. Male
    b. Female
    c. Other
2. **What is your current age? [C]**

    ____
3. **Please specify your ancestry (tick up to two only) [C]**
    a. Australian
    b. Aboriginal
    c. Torres Strait Islander
    d. Chinese
    e. English
    f. Filipino
    g. German
    h. Irish
    i. Indian
    j. Italian
    k. Scottish
    l. Other ancestry 1 (please specify below)
        i. ___________________________________
    m. Other ancestry 2 (please specify below)
        i. ___________________________________
4. **What is your religion? [C]**
    a. Christianity/Catholicism
    b. Judaism
    c. Islam
    d. Buddhism
    e. Hinduism
    f. No religion
    g. Other (please specify below)
        i. ___________________________________
5. **What is the highest level of education you have completed? [C]**
    a. Less than high school
    b. High school
    c. Bachelor's degree
    d. Master's degree
    e. PhD or higher
    f. Trade school
6. **What is your weekly household income? [C]**
    a. $1–500 per week
    b. $501–1000 per week
    c. $1001–1500 per week
    d. $1501–2000 per week
    e. $2001–2500 per week
    f. >$2500 per week

KEY

C: Conditional Influences; S: Social Influences; M: Motivational Influences; P: Practical Influences; B: Benefits; H: Harms; T: Trust.

7. **Do you have any of the following conditions? (tick all that apply) [C]**
    a. Asthma
    b. Cancer
    c. Current smoker
    d. Chronic kidney disease
    e. Chronic obstructive pulmonary disorder
    f. Cystic fibrosis
    g. Diabetes
    h. Down syndrome
    i. Heart disease (e.g. heart failure, coronary artery disease, cardiomyopathies)
    j. HIV
    k. Hypertension
    l. Immunocompromised or immunosuppressed
    m. Liver disease
    n. Neurological conditions (e.g. Alzheimer's disease, dementia)
    o. Obesity
    p. Other lung diseases (e.g. interstitial lung disease, pulmonary fibrosis, pulmonary hypertension)
    q. Previous stroke
    r. Sickle cell disease
    s. Solid organ or blood stem cell transplantation
    t. Substance use disorders
    u. Thalassemia
    v. None
8. **Has a doctor or other healthcare professional ever recommended that you get a COVID-19 vaccine? [S]**
    a. No
    b. Yes
9. **Where do you receive your news from? (tick all that apply) [S]**
    a. Community/religious leaders
    b. Online news articles
    c. Print news (e.g. newspapers, magazines)
    d. Radio
    e. Social media
    f. Television news broadcast
    g. Word of mouth
    h. Other (please specify below)
        i. ________________________________
10. **Have you seen or heard any information about COVID-19 vaccines (e.g., on the news, on social media, or from friends and family) that you could not determine were true or false? [S]**
    a. No
    b. Yes
11. **Select your top 3 most trusted sources of information about COVID-19 vaccines: [S]**
    a. Australian Department of Health
    b. Employer
    c. General practitioner

d. Hospital system websites (e.g. Blacktown & Mount Druitt Hospital website)
e. News sources (e.g. television, internet, radio)
f. Nurses
g. NSW Health
h. Online publishers of medical information (e.g. WebMD or Mayo Clinic)
i. Pharmacists
j. Professional organisation(s)
k. Religious Leader(s)
l. Social media (e.g. Facebook, Twitter, Instagram, WhatsApp, LinkedIn, Tik-Tok)
m. Therapeutic Goods Administration
n. Union leader(s)
o. Other (please specify below)
    i. ___________________________________

12. **Have you or anyone in your family or friends had COVID-19?** **[S]**
    a. No
    b. Yes
13. **What motivated you to get vaccinated? (tick all that apply)** **[M]**
    a. Protect my health
    b. Protect the health of family/friends
    c. Protect the health of colleagues
    d. Protect the health of my community
    e. To get back to work/school
    f. To resume social activities
    g. To resume travel
    h. Because others encouraged me to get vaccinated (please specify below)
        i. ___________________________________
    i. Other (please specify below)
        i. ___________________________________
    j. Not sure
14. **In terms of accessibility, how difficult was it for you to get a COVID-19 vaccine?** **[P]**
    a. Not at all difficult
    b. A little difficult
    c. Neutral
    d. Somewhat difficult
    e. Very difficult

Vaccine confidence scale

How much do you agree or disagree with the following statements?

15. ***COVID-19 vaccines are necessary to protect your own health*** **[B]**
    a. Strongly disagree
    b. Disagree
    c. Neither agree nor disagree
    d. Agree
    e. Strongly Agree

KEY
C: Conditional Influences; S: Social Influences; M: Motivational Influences; P: Practical Influences; B: Benefits; H: Harms; T: Trust.

16. ***COVID-19 vaccines do a good job in preventing the diseases they are intended to prevent* [B]**
    a. Strongly disagree
    b. Disagree
    c. Neither agree nor disagree
    d. Agree
    e. Strongly Agree
17. ***COVID-19 vaccines are safe* [B]**
    a. Strongly disagree
    b. Disagree
    c. Neither agree nor disagree
    d. Agree
    e. Strongly Agree
18. ***If I do not vaccinate myself, I may get COVID-19 and cause others in the community to also get the disease* [B]**
    a. Strongly disagree
    b. Disagree
    c. Neither agree nor disagree
    d. Agree
    e. Strongly Agree
19. ***I am concerned about getting COVID-19* [H]**
    a. Strongly disagree
    b. Disagree
    c. Neither agree nor disagree
    d. Agree
    e. Strongly Agree
20. ***The risk of serious side effects from COVID-19 vaccines (e.g. hospitalisation) is higher than the risk of contracting the COVID-19 disease* [H]**
    a. Strongly disagree
    b. Disagree
    c. Neither agree nor disagree
    d. Agree
    e. Strongly Agree
21. ***I have a good relationship with my general practitioner* [T]**
    a. Strongly disagree
    b. Disagree
    c. Neither agree nor disagree
    d. Agree
    e. Strongly Agree
22. ***In general, the government and public health agencies in charge of COVID-19 vaccinations have my best interests in mind* [T]**
    a. Strongly disagree
    b. Disagree
    c. Neither agree nor disagree
    d. Agree
    e. Strongly Agree

KEY
C: Conditional Influences; S: Social Influences; M: Motivational Influences; P: Practical Influences; B: Benefits; H: Harms; T: Trust.
